# Impact of Breakfast Skipping and Breakfast Choice on the Nutrient Intake and Body Mass Index of Australian Children

**DOI:** 10.3390/nu8080487

**Published:** 2016-08-10

**Authors:** Flavia Fayet-Moore, Jean Kim, Nilani Sritharan, Peter Petocz

**Affiliations:** 1 Nutrition Research Australia, Level 13 167 Macquarie Street, Sydney 2000, NSW, Australia; 2 Nestlé Australia, 1 Homebush Bay Drive, Rhodes 2138, NSW, Australia; jean.kim@au.nestle.com; 3 Cereal Partners Worldwide, Chemin du Viaduc 1, Prilly 1008, Vaud, Switzerland; nilani.sritharan@nestle.com; 4 Department of Statistics, Macquarie University, Sydney 2109, NSW, Australia; peter.petocz@mq.edu.au

**Keywords:** breakfast, children, cereal, BMI, nutrient, fibre, folate, micronutrient, National Nutrition Survey, overweight

## Abstract

Recent data on breakfast consumption among Australian children are limited. This study examined the impact of breakfast skipping and breakfast type (cereal or non-cereal) on nutrient intakes, likelihood of meeting nutrient targets and anthropometric measures. A secondary analysis of two 24-h recall data from the 2007 Australian National Children’s Nutrition and Physical Activity Survey was conducted (2–16 years; *n* = 4487) to identify (a) breakfast skippers and (b) breakfast consumers, with breakfast consumers further sub-divided into (i) non-cereal and (ii) cereal consumers. Only 4% skipped breakfast and 59% of skippers were 14–16 years. Breakfast consumers had significantly higher intakes of calcium and folate, and significantly lower intakes of total fat than breakfast skippers. Cereal consumers were more likely to meet targets and consume significantly higher fibre, calcium, iron, had significantly higher intakes of folate, total sugars and carbohydrate, and significantly lower intakes of total fat and sodium than non-cereal consumers. The prevalence of overweight was lower among breakfast consumers compared to skippers, and among cereal consumers compared to-cereal consumers (*p* < 0.001), while no significant differences were observed for mean body mass index (BMI), BMI *z*-score, waist circumference and physical activity level across the categories. Breakfast and particularly breakfast cereal consumption contributes important nutrients to children’s diets.

## 1. Introduction

Breakfast is often referred to as one of the most important meals of the day, helping kick start metabolism and re-fuelling the body with energy and essential nutrients [1,2,3,4]. A review on breakfast habits and academic performance reported that children who consumed breakfast had higher daily nutrient intakes, were more likely to consume healthier diets and made better food choices, than children who skipped breakfast [3]. Studies of adolescent children in the both US and Australia have shown that breakfast consumption is associated with lower body mass index (BMI) [5,6], and in Canada it has been estimated that not eating breakfast every day nearly doubled the odds of being overweight at 4.5 years of age [6,7]. A recent meta-analysis found that breakfast skipping is associated with an increased risk of developing type 2 diabetes [8]. In Australia, childhood breakfast skipping was associated with both higher waist circumference and negative effects on cardio-metabolic health markers in adulthood [9].

Not only having breakfast, but type of breakfast may be important for meeting nutrient targets. A large body of epidemiological research report that children who have breakfast cereal for breakfast are more likely to meet their recommended intakes of B vitamins (niacin, thiamine, folate), calcium, iron and fibre [4,10,11,12,13,14,15]. In a recent systematic review, breakfast cereal consumption was associated with diets higher in vitamins and minerals, higher Healthy Eating Index, and was not associated with increased total energy or sodium intakes [16]. In addition, the review showed that among children, regular consumption of breakfast cereals was associated with a lower BMI and lower risk of being overweight or obese.

In the 1995, Australian National Nutrition Survey, breakfast cereal and milk consumption combined provided >25% of the Recommended Dietary Intake (RDI) for several nutrients including B vitamins, iron and calcium [17]. More recently, Grieger and Cobiac 2012 compared nutrient intakes according to breakfast choice in a sample of Australian boys aged 14–16 years and found that ready-to-eat cereal consumers had a diet with greater nutrient density intake compared to non-cereal consumers and breakfast skippers, as well as a lower BMI and waist circumference [12].

As the most recent data on the breakfast consumption habits of a nationally representative sample of Australian children and adolescents are nearly 20 years old, the aim of this study was to investigate breakfast consumption, in particular breakfast choice (cereal vs. non-cereal), and its association with nutrient intake, likelihood of meeting nutrient targets, and anthropometric measures.

## 2. Materials and Methods

### 2.1. Survey Methodology

Data from the 2007 Australian National Children’s Nutrition and Physical Activity Survey (ANCNPAS) were utilised and consisted of a randomly selected representative sample of the Australian population, containing 4487 children aged 2 to 16 years old. Survey data were collected by the Commonwealth Scientific and Industrial Research Organisation (CSIRO) and the University of South Australia, and permission to access the data was granted by the Australian Social Sciences Data Archives [18]. Full details of the ANCNPAS methodology are contained in the User’s Guide [19]. Ethics approval for the ANCNPAS was obtained from the National Health and Medical Research Council registered Ethics Committees of CSIRO and University of South Australia. Food, beverage, and supplement intakes were collected for all participants using a standardised, computer-based, 24-h recall methodology with a food model booklet to estimate portion sizes. Data were collected on two days from participants or their caregivers between February and August, 2007. A computer-assisted personal interview was conducted in the child’s home, and this was followed 7 to 21 days later by a computer-assisted telephone interview. Physical activity was measured by “use of time” among children aged 9 to 16 years old via a validated computerised 24-h recall methodology [20]. Physical activity level, which is a multiple of resting metabolic rate was calculated by multiplying the estimates of activity-specific energy expenditure by the number of minutes reported for each activity, and averaging across the 1440 m of each day. Physically active and sedentary behaviours collected in the survey included number of minutes per day undertaking moderate to vigorous physical activity (at least 3 METs), vigorous activity (at least 6 METs), organised sport and play, free play, active transport, out of school hours screen time, total screen time, television, video game, computer use, passive transport, non-screen sedentary behaviour and sleep. Weight, height, and waist circumference were measured, and body mass index was calculated as weight (kg)/height (m^2^). BMI *z*-score [21] or centile adjusted for age and sex was calculated using the US CDC 2000 growth reference chart [22].

### 2.2. Dietary Assessment

Breakfast was defined as a caloric intake between 5:00 a.m. and 9:30 a.m., which is based on the time period where the first peak of percent energy intake occurs for this population [23]. Breakfast consumers were children who consumed an energy containing food or beverage at breakfast and breakfast skippers were those who did not (Table 1). Breakfast cereal was defined as: Ready-to-eat cereals, puffed grains, muesli, oats (natural and as porridge/oatmeal), and semolina. Breakfast cereal portion size was calculated as the amount of breakfast cereal consumed, in grams, during breakfast. Where oats were reported as consumed with milk and/or water, grams of oats consumed was estimated based on the fibre content of the breakfast cereal as consumed.

### 2.3. Statistical Analysis

Mean daily energy, nutrient intake, contribution of likelihood of meeting the estimated average requirement (EAR) for fibre, calcium, iron, and BMI, waist circumference and physical activity level (PAL) were compared between breakfast consumers and skippers and between breakfast cereal consumers and non-cereal breakfast consumers. General linear regression models included age, sex, energy intake and physical activity level as covariates, and comparisons between categories of breakfast and cereal consumption were made by ANOVA models. Statistical significance was set at *p* < 0.01.

## 3. Results

### 3.1. Breakfast and Cereal Consumption

Of a total of 4487 children surveyed, only 4% (*n* = 198) were breakfast skippers (Table 2). Breakfast skipping increased with age, and the highest proportion of breakfast skipping occurred among children aged 14–16 years (59.1%). Skippers were significantly older (12.8 ± 0.3 years) than breakfast consumers (8.4 ± 0.1 years) *p* < 0.0001) and there were significantly more girls (61%, *n* = 121) compared to boys (39%, *n* = 77, *p* < 0.001).

Gender and age trends for non-cereal consumers were similar to those of breakfast skippers. The prevalence of having a non-cereal breakfast increased significantly with age for both boys and girls (*p* < 0.0001). Non-cereal consumers were older (9.8 ± 0.1 years) compared to cereal consumers (7.6 ± 0.1 years, *p* < 0.0001) and there were significantly more girls (55%, *n* = 801) compared to boys (45%, *n* = 644) (*p* < 0.0001). The highest prevalence of both breakfast and cereal consumption was among children aged 4–8 years. The average portion size of cereal consumed by boys (36.1 ± 0.8 g) was significantly higher than that consumed by girls (30.4 ± 0.9 g, *p* < 0.0001). Cereal portion size was significantly greater among older children: 9–13 years (38.0 ± 1.2 g) and 14-16 years (34.6 ± 1.6 g) than among those aged 2–3 years (29.1 ± 1.3 g) and 4–8 years (32.0 ± 1.1 g).

### 3.2. Energy and Nutrient Intakes

Total daily energy intake across all breakfast categories were similar (Table 3). Breakfast consumers had significantly higher adjusted mean daily intake of calcium and folate and significantly lower total fat compared to breakfast skippers. The type of breakfast (non-cereal or cereal) did not influence total daily energy, protein, saturated fat, riboflavin, niacin or thiamine intakes. However, cereal consumers had a significantly higher mean daily intake of total carbohydrates including sugars and fibre, calcium, iron and folate, and lower total fat and sodium intakes than non-cereal consumers. The higher micronutrient intakes among breakfast and cereal consumers resulted in greater likelihood of meeting nutrient targets. A large proportion of breakfast skippers did not meet the Estimated Average Requirement (EAR) for calcium (74.2%) and the Adequate Intake (AI) for fibre (77.3%) and they were less likely to meet the EAR for iron compared to breakfast consumers (Table 4). In contrast, a large proportion of breakfast consumers met the EAR for calcium (98.2%) and iron (96.7%), and the AI for fibre (97.9%), and these were significantly higher than for breakfast skippers. Breakfast consumers were 5.5 times more likely to meet the EAR for calcium; 3.3 times more likely to meet the AI for fibre and 8.0 times more likely to meet the EAR for iron (Chi-Square *p* < 0.01) than breakfast skippers. Type of breakfast resulted in differences in the proportion of children who met nutrient targets. Cereal consumers were 3.0 times more likely to meet the EAR for calcium; 1.6 times more likely to meet the AI for fibre and 7 times more likely to meet the EAR for iron compared to non-cereal consumers (*p* < 0.0001).

### 3.3. Anthropometric Measures

There were no significant differences in mean body mass index (BMI), BMI *z*-score, waist circumference and physical activity level across the four categories. In contrast, the prevalence for overweight and obesity among breakfast consumers was significantly lower (overweight: 16.4%; obese: 16.5%, Chi-Square *p* < 0.001) than breakfast skippers (overweight: 21.2%; obese: 23.2%, Chi-Square *p* < 0.001). Cereal consumers had a lower prevalence of overweight and obesity (overweight: 16.2%; obese: 15.4%) than non-cereal breakfast consumers (overweight: 16.7%; obese: 18.8%) (Table 2).

## 4. Discussion

The majority of Australian children and adolescents consumed breakfast, over two-thirds had cereal during breakfast and both breakfast and cereal consumers were more likely to meet targets for calcium and fibre compared to breakfast skippers and non-cereal consumers. There was no difference in total energy, BMI *z*-score, waist circumference or physical activity level across breakfast categories, and both breakfast and cereal consumers had a lower prevalence of overweight and obesity than skippers and non-cereal breakfast consumers, respectively.

### 4.1. Breakfast Consumption

The most conclusive benefit of breakfast consumption in the literature is the contribution to total nutrient intakes [3]. Hence, breakfast skipping among children and adolescents may be a concern because of the missed opportunity for a nutrient-rich eating occasion. In our study, prevalence of breakfast skipping was low. In Australia, breakfast skipping ranges from 4% to 30% depending on the population studied and the definition used to classify respondents as breakfast consumers [12,24,25,26]. Although few Australian children skipped breakfast, the breakfast meal contributes proportionally more to total energy intake in younger children compared to those aged 16–18, specifically among girls [17]. In the US, breakfast skipping has increased over time [27,28]. A review of breakfast habits reported that the prevalence of skipping ranged between 10% and 30% [29]; while other studies report skipping as low as 4% among children 9–14 years [30] and as high as 57% in some ethnic groups [31]. In our study, the highest prevalence of skippers were among adolescent females. Strategies to promote breakfast among adolescent females may be beneficial, as several studies show that breakfast consumption is dependent on age and gender—particularly among adolescents, breakfast skipping is not only common [32], but it is the most skipped of all meals [33]. In Canada, 10% of children and adolescents were breakfast skippers, but the highest proportion of skippers (18%) was also among those aged 14–18 years [34]. Several other studies have found that the prevalence of skipping increases with age [5,15,29,35] and that girls are more likely to skip breakfast than boys [24,30,32,36,37,38]. Thus, interventions to improve dietary patterns of adolescents could focus on the breakfast meal.

### 4.2. Energy and Nutrient Intakes

We found that total daily energy intake was not significantly different across breakfast categories. Greater breakfast and cereal consumption is usually associated with greater reported energy intakes compared to lower cereal consumption and breakfast skipping [39]. Many studies show that breakfast cereal consumers have higher energy intakes, however few studies adjust energy intakes for age [39,40]—as we have done. The lower reported energy intake among skippers in these studies may also be due to deliberate underreporting of energy due to dieting, where energy is restricted by skipping breakfast [40]. Despite similar energy intakes, skippers had lower nutrient intakes. This may be because the missed nutrients from skipping breakfast are not usually compensated for during other meals [41].

Breakfast choice may positively or negatively affect breakfast quality and as with any eating occasion, it provides an opportunity to improve nutrient intakes. Proposed guidelines for high-quality breakfast include fibre-rich wholegrains, fruit, low-fat dairy products, lean protein and healthy fats [42]. In line with our findings, recent reviews report that breakfast [43] and cereal for breakfast [3,4,16,44] are associated with higher nutrient intakes, and that cereal consumers in particular, have a more favourable nutrient profile. In a recent review, 30 cross-sectional studies among children and adolescents consistently reported that children who regularly consume breakfast cereals have diets higher in percent energy from carbohydrate, total sugars, dietary fibre, but also key micronutrients including folate, calcium and zinc [16]. Australian data show that breakfast and breakfast cereals are a good source of B vitamins, vitamin C, calcium and iron [17] and contribute less than 10% of the daily added sugar intake among children aged 2–18 years [45]. Greater total daily nutrient intakes among cereal consumers may be explained by the nutrient composition of the breakfast cereal. Many ready-to-eat cereals are fortified with vitamins and minerals such as iron and folate [11,46,47] thus contributing to higher total daily nutrient intakes [48] and consequently to greater nutrient status, such as folate [11]. The differences in daily intakes across breakfast and cereal groups may also reflect other foods consumed throughout the day, as breakfast consumers have been shown to make healthier food choices [3]. Hence, further research on the food choices that non-cereal consumers make, could be useful to understand differences in micronutrient intakes associated with breakfast choice.

We found greater intakes of calcium and folate, lower intakes of sodium and a greater likelihood of meeting calcium, fibre and iron targets were among breakfast and cereal consumers compared to skippers and non-cereal consumers. Cereal is a driver for dairy consumption [1,11,12,13,14,29,30,49,50,51,52,53,54,55]. A third of total daily milk was consumed with breakfast cereal in this survey [56] and in the US, virtually all cereal consumers have milk with cereal [55]. Fortification of breakfast cereals resulted in greater calcium intakes in a randomized trial among US children [57]. Fibre intakes have been shown to be higher among children and adolescents who consume a ready-to-eat cereal at breakfast compared to a non-cereal breakfast [10,16,29,58]. Where a difference is not observed, it is often related to the study’s definition of breakfast cereal; and may exclude other cereals that are good sources of fibre, such as muesli and oats. It may be useful for future studies to investigate the type of cereal (ready-to-eat vs. muesli vs. porridge/oatmeal) and its effect on nutrient intakes, as not all cereals can be good sources of fibre and micronutrients; and not all cereals are fortified with micronutrients. Not only is having a cereal for breakfast important to consider for meeting nutrient targets, but the amount is too. The relationship between nutrient intakes and meeting nutrient recommendations is directly related to the amount and frequency of breakfast cereal consumed [11,15,49]. The mean portion of breakfast cereal consumed across all ages in this study were less than 40 g per day, consistent with the Australian Dietary Guidelines recommendations of a serve of grain (cereal) food, which is 30 g for wheat cereal flakes and muesli [59]. We found that boys and older children had the largest portion of breakfast cereal and that this finding is consistent with the UK [11] and the US [3].

### 4.3. Anthropometry

We found no differences in anthropometric measures across the breakfast categories for mean BMI, BMI z-score, waist circumference and physical activity levels. In contrast, both breakfast and cereal consumers were less likely to be overweight or obese compared to skippers and non-cereal consumers, respectively. Despite the well documented evidence for an association between breakfast and overweight [29,60,61], the mechanisms linking, consumption in general and breakfast cereal consumption, in particular, and their association with overweight and obesity are not clearly understood. As with our study, the literature is predominantly of a cross-sectional nature [62]. The association between breakfast and prevalence of overweight and obesity in our study could be driven by the association between cereal consumption and overweight, as a large proportion of children that had breakfast, had cereal (66%). In a prospective study of girls who were 9 years at baseline, the authors reported that the inverse association between breakfast and weight status was no longer significant when breakfast cereal consumption was adjusted for [50]. A recent systematic review on breakfast cereal [16] concluded that regular breakfast cereal consumption is associated with a lower body mass index and lower risk of being overweight or obese. The review included the only meta-analysis [39] and a prospective cohort [50] on the subject. Although a proposed mechanism for breakfast and breakfast cereal’s role in weight management is needed, greater milk intakes reported with cereal may be a contributing factor, as greater dairy intakes have been inversely associated with weight status [63,64]. It is necessary to investigate the impact of breakfast choice and perhaps type of cereal (higher sugar versus lower sugar, high fibre, wholegrain), on anthropometric measures in longer-term trials. Another possible explanation for the association with lower overweight and obesity reported in our study and in the literature may be due to confounding factors such as the timing of food consumption, or eating patterns of breakfast and cereal consumers that may be more favourable for the regulation of body weight [65,66]. For example, timing of food intake can influence energy regulation and reduce risk of weight gain [65]. Further, reverse causality may be a confounder, with breakfast and cereal consumption as markers for other healthful lifestyle factors among children that may have a greater influence on weight regulation than the breakfast or cereal per se [67]. Eating breakfast may also reflect better lifestyles and food choices across the day, explaining some of the limited association we observed with anthropometric measures. Skipping breakfast has been associated with health compromising behaviours among adolescents [36], with lower physical activity levels [10], and poorer food choices [3].

This study has major strengths including the use of a large sample of nationally representative children and adolescents, the comparison of categories of breakfast consumption based on cereal vs. non cereal, as well as the adjustment for confounders including physical activity. It is important to note the limitations of a cross-sectional analysis, where the results are limited to the description of relationships rather than causal associations. Since the data were only collected on two days, it is not known whether breakfast cereal consumption is a regular occurrence. However, we defined breakfast consumers and skippers based on two recall days to better reflect dietary patterns that may persist over time. There are inherent limitations with any dietary recall methodology, including its dependence on memory, which can lead to over- or under-reporting. Under-reporting in the 2007 Australian National Children’s Nutrition and Physical Activity Survey ranged from 6.0% to 6.7% and over-reporting ranged from 1.6% to 3.0% [68]. Prevalence of skipping is difficult to compare across studies due to differences in definitions used and study designs. Breakfast consumption defined as having something to eat during breakfast time using a 24-h dietary recall may not reflect usual breakfast consumption habits.

## 5. Conclusions

We examined the breakfast consumption habits of Australian children and adolescents and showed that children who had breakfast, and in particular cereal, had better total dietary intake profiles, including intakes of key nutrients, such as fibre, calcium and folate and were more likely to be of normal weight, despite no differences in energy intake, BMI, or physical activity level. Additional clinical trials investigating specific foods and beverages consumed at breakfast, timing of meals and how those choices impact on weight status are warranted.

## Figures and Tables

**Table 1:** Breakfast category definitions.

Breakfast Category	Definition
1. Breakfast skippers	Children who did not consume an energy containing food or beverage during breakfast (5:00 a.m.–9:30 a.m.) on both recall days.
2. Breakfast consumers	Children who consumed an energy containing food or beverage during breakfast on one of the recall days.
(i) Cereal consumers	Children who consumed cereal at breakfast.
(ii) Non-cereal consumers	Children who did not consume cereal at breakfast, where intake was 0 g.

**Table 2:** Descriptive analysis of children and adolescents by breakfast categories (mean ± SE; % within each category).

	Breakfast		Breakfast Consumers	
	Skippers	Consumers	Non-Cereal	Cereal
Age (2–16 years)	*n* = 198 12.8 ± 0.3 (4%)	*n* = 4289 8.4 ± 0.1 * (96%)	*n* = 1445 9.8 ± 0.1 (34%)	*n* = 2844 7.6 ± 0.1 * (66%)
Boys *n* (%)	77 (38.9%)	2172 (50.6%)	644 (44.6%)	1528 (53.7%)
Girls *n* (%)	121 (61.1%) *	2117 (49.4%)	801 (55.4%) *	1316 (46.3%)
Physical activity level	1.68 ± 10.03	1.65 ± 0.01	1.65 ± 0.01	1.65 ± 0.01
TV minutes	160 ± 9.8	154 ± 1.9	155 ± 3.1	153 ± 3.1
BMI ^1^ (kg/m^2^)	18.6 ± 0.4	18.5 ± 0.1	18.7 ± 0.1	18.4 ± 0.1
BMI *z*-score ^1^	0.54 ± 0.1	0.62 ± 0.2	0.69 ± 0.04	0.58 ± 0.03
Waist circumference ^1^ (cm)	61.9 ± 0.9	62.3 ± 0.2	62.4 ± 0.3	61.7 ± 0.2
% Normal weight	55.6	67.1 *	64.5	68.4 *
% Overweight	21.2 *	16.4	16.7 *	16.2
% Obese	23.2 *	16.5	18.8 *	15.4

* Chi-square comparison between breakfast skippers vs. consumers, and non-cereal vs. cereal breakfast consumers, *p* < 0.001; ^1^ Adjusted for age, gender, total daily energy intake and physical activity level.

**Table 3:** Total daily energy and nutrient intake by breakfast categories.

	Breakfast		Breakfast Consumers	
Energy and Nutrient Intakes *	Skippers	Consumers	Non-Cereal Consumers	Cereal Consumers
Energy ^1^ (kJ/day)	7742 ± 268	7813 ± 50.8	7753 ± 85.4	7816 ± 62.2
Protein ^2^ (g/day)	75.9 ± 2.7	80.1 ± 0.5	78.7 ± 0.9	80.5 ± 0.6
Total fat (g/day)	71.7 ± 1.3	66.9 ± 0.3 *	68.5 ± 0.4	65.8 ± 0.3 *
Saturated fat (g/day)	31.7 ± 0.8	30.2 ± 0.2	30.3 ± 0.3	30.0 ± 0.2
Carbohydrate (g/day)	234 ± 3.5	241 ± 0.7	237 ± 1.1	242 ± 0.8 *
Total Sugars (g/day)	112.0 ± 3.4	118.8 ± 0.7	116 ± 1.1	120 ± 0.8 *
Fibre (g/day)	18.5 ± 0.7	20.2 ± 0.1	19.4 ± 0.2	20.5 ± 0.2 *
Calcium (mg/day)	749 ± 32	861 ± 6.1 *	774 ± 10.2	905 ± 7.5 *
Iron (mg/day)	10.0 ± 0.6	11.2 ± 0.1	10.1 ± 0.2	11.8 ± 0.1 *
Niacin (mg/day)	38.3 ± 3.0	43.9 ± 0.6	42.1 ± 1.0	44.7 ± 0.7
Thiamine (mg/day)	1.58 ± 0.6	2.2 ± 0.1	2.1 ± 0.2	2.2 ± 0.1
Riboflavin (mg/day)	2.30 ± 0.8	3.11 ± 0.2	2.9 ± 0.3	3.2 ± 0.2
Folate (mg/day)	379 ± 30.7	491 ± 5.8 *	462 ± 10	506 ± 7.3 *
Vitamin C (mg/day)	98.0 ± 18.2	124 ± 3.6	127 ± 6.0	121 ± 4.3
Vitamin A (retinol equivalents μg/day)	711 ± 100	799 ± 20	808 ± 33	790 ± 24
Vitamin E (a-tocopherol equivalents mg/day)	6.0 ± 1.0	6.6 ± 0.2	7.0 ± 0.3	6.3 ± 0.2
Phsphorus (mg/day)	1304 ± 29	1354 ± 5.7	1301 ± 9.4	1380 ± 6.8 *
Magnesium (mg/day)	256 ± 7.3	279 ± 1.5 *	268 ± 2.4	285 ± 1.7 *
Potassium (mg/day)	2534 ± 69	2680 ± 14	2620 ± 23	2704 ± 16 *
Zinc (mg/day)	8.8 ± 0.4	10.3 ± 0.1 *	9.6 ± 0.1	10.6 ± 0.1 *
Iodine (µg/day)	121 ± 5.7	133 ± 1.1	122 ± 1.8	139 ± 1.3 *
Sodium (mg/day)	2581 ± 80.9	2374 ± 15.4	2482 ± 25	2311 ± 19 *

* Denotes significant differences between breakfast skippers and consumers, and between non-cereal and cereal breakfast consumers, *p* < 0.01; ^1^ Adjusted age, gender and physical activity level; ^2^ Adjusted for age, gender, energy intake and physical activity level.

**Table 4:** Likelihood of meeting the estimated average requirement (EAR) for calcium and iron, and the adequate intake (AI) for fibre.

Nutrient	Meeting EAR/AI	Breakfast		Breakfast Consumers	
		Skippers *n* (%)	Consumers *n* (%)	Non-Cereal *n* (%)	Cereal *n* (%)
Calcium	Yes	51 (25.8%)	2821 (65.8%)	706 (48.9%)	2115 (74.4%)
	No	147 (74.2%)	1468 (34.2%)	739 (51.1%)	729 (25.6%)
Fibre	Yes	45 (22.7%)	2116 (49.3%)	597 (41.3%)	1519 (53.4%)
	No	153 (77.3%)	2173 (50.7%)	848 (58.7%)	1325 (46.6%)
Iron	Yes	141 (71.2%)	4074 (95.0%)	1281 (88.7%)	2793 (98.2%)
	No	57 (28.8%)	215 (5.0%)	164 (11.3%)	51 (1.8%)

*p* < 0.001, breakfast skippers vs. consumers; non-cereal vs. cereal breakfast.; EAR—estimated average requirement; AI—adequate intake.
